# Predicting anti-cancer drug combination responses with a temporal cell state network model

**DOI:** 10.1371/journal.pcbi.1011082

**Published:** 2023-05-01

**Authors:** Deepraj Sarmah, Wesley O. Meredith, Ian K. Weber, Madison R. Price, Marc R. Birtwistle

**Affiliations:** 1 Department of Chemical and Biomolecular Engineering, Clemson University, Clemson, South Carolina, United States of America; 2 The University of Virginia School of Medicine, Charlottesville, Virginia, United States of America; 3 College of Pharmacy, Medical University of South Carolina, Charleston, South Carolina, United States of America; 4 Department of Bioengineering, Clemson University, Clemson, South Carolina, United States of America; Pázmány Péter Catholic University: Pazmany Peter Katolikus Egyetem, HUNGARY

## Abstract

Cancer chemotherapy combines multiple drugs, but predicting the effects of drug combinations on cancer cell proliferation remains challenging, even for simple *in vitro* systems. We hypothesized that by combining knowledge of single drug dose responses and cell state transition network dynamics, we could predict how a population of cancer cells will respond to drug combinations. We tested this hypothesis here using three targeted inhibitors of different cell cycle states in two different cell lines *in vitro*. We formulated a Markov model to capture temporal cell state transitions between different cell cycle phases, with single drug data constraining how drug doses affect transition rates. This model was able to predict the landscape of all three different pairwise drug combinations across all dose ranges for both cell lines with no additional data. While further application to different cell lines, more drugs, additional cell state networks, and more complex co-culture or *in vivo* systems remain, this work demonstrates how currently available or attainable information could be sufficient for prediction of drug combination response for single cell lines *in vitro*.

## Author summary

Cancer chemotherapy combines multiple drugs, but determining which drugs would be efficacious for particular patients remains extremely challenging. Experimental solutions to this problem are not yet possible due to the large space of possible combinations of hundreds of anti-cancer drugs. Computational models may help, but it is not yet clear how such models should be built and what elements they need to capture to predict drug combination response. In this work, we explored the idea that if we knew something about the proportions of different types of cancer cells in a population, how fast transitions happen between the different types, and how individual drugs affect those transitions, that we might be able to build a computational model that predicts drug combination responses based only on feasible single drug response experiments. We tested this idea using simple cell culture systems with two different cancer cell lines and three different anti-cancer drugs, and found surprisingly good agreement between computational model predictions and experimentally measured drug combination responses. While further application to different cell lines, more drugs, and more complex experimental systems remain, this work demonstrates how currently available or attainable information could be sufficient for prediction of drug combination responses.

## Introduction

Matching chemotherapy regimens to cancer patients remains a grand challenge of oncology and personalized medicine. Targeted drugs often have genetic biomarkers, such as BRAFV600E for vemurafenib [1,2], EGFR mutations and copy number amplification for gefitinib [3–5], BCR-ABL fusion for imatinib [6,7], and HER2 copy number amplification for trastuzumab [8,9]. However, such matched patients often do not respond to therapy and/or eventually develop resistance. Why? One major driver is tumor heterogeneity; cells in different “states” that have different drug sensitivities. Genomic status (and mutational heterogeneity across a population)[10] is one dimension of cell state, but cell states are also defined by their histology or transcriptomics (through for example single cell RNAseq experiments) [11–17], and it is becoming appreciated that cells can transition between such states in development-like networks, sometimes called cell state networks [13,18]. Such plasticity between cell states can contribute to drug resistance [19–21], and combinations of drugs targeting different pathways and factors involving phenotype transition have been proposed to prevent such resistance [19]. Another is the multi-variate complexity of biochemical networks within which drug targets reside and by which chemotherapy drugs exert their action [22–27]. These networks can differ between cell states, adapt to therapy, and also give rise to non-intuitive therapy results, such as feedback loops and compensatory pathways underlying the efficacy of combining Raf and MEK inhibitor combinations, which lie in the same genetic pathway [28–31].

Massive agnostic efforts have screened thousands of cancer cell lines for sensitivity to hundreds of anti-cancer drugs, with matched multi-omic data to mine for biomarkers predictive of drug response [32–39]. These efforts, while substantial, still have not solved the problem of how to match patients to drugs. Moreover, many chemotherapy regimens comprise combinations of 3–4 drugs. Comprehensive experimental exploration of just 2-way drug combinations for hundreds of anti-cancer drugs across a representative cohort is infeasible clinically, and currently unreachable even in cell culture systems.

The inability to obtain an experimental solution to the problem of matching drug combinations to patients has motivated computational modeling approaches. In principle, more comprehensive exploration of drug combination space could be achieved *in silico*. Various computational methods including mechanistic models and machine learning approaches have shown promise in predicting drug combination responses, especially taking into consideration context specific pathology and omics data as well as identifying specific biomarkers and drug-targets [40–45]. Regardless of the modeling methods being used, there is a widespread focus on using information about biochemical networks to facilitate drug combination response prediction [27,46–48]. Despite advanced methods being applied to integrate such information into models, building predictive drug combination response models remains an unsolved challenge. Any solution to this problem must invariably rely on experimental data that is already existing or is realistically attainable, such as single drug dose responses.

In this paper, rather than focus on modeling biochemical networks, we test the hypothesis that by combining knowledge of single drug dose responses and cell state transition network dynamics, we could predict how a population of cancer cells will respond to drug combinations. Although this hypothesis runs contrary to the predominant biochemical network-centered view of this problem, cell state transitions are largely governed by biochemical networks in which drug targets are embedded, so in a sense this idea is encompassing prior logic. We test this hypothesis by focusing on three drugs that target cell cycle transitions in two different cell lines *in vitro*. A Markov model is developed to capture population growth dynamics and single drug dose responses, and then this model is used to predict all two-way drug combination responses with no further adjustment. Comparison of these model predictions to experimental tests shows surprisingly good agreement, despite the simplicity of the model. These results suggest a sufficient formulation for predicting how single cell line population growth dynamics *in vitro* respond to drug combinations, relying only on currently available and/or attainable information. If this idea scales upon more extensive testing with additional cell lines, drugs, and more complex biological scenarios such as co-culture or *in vivo* systems it could have widespread impact on precision oncology efforts.

## Results

**Fig 1:**
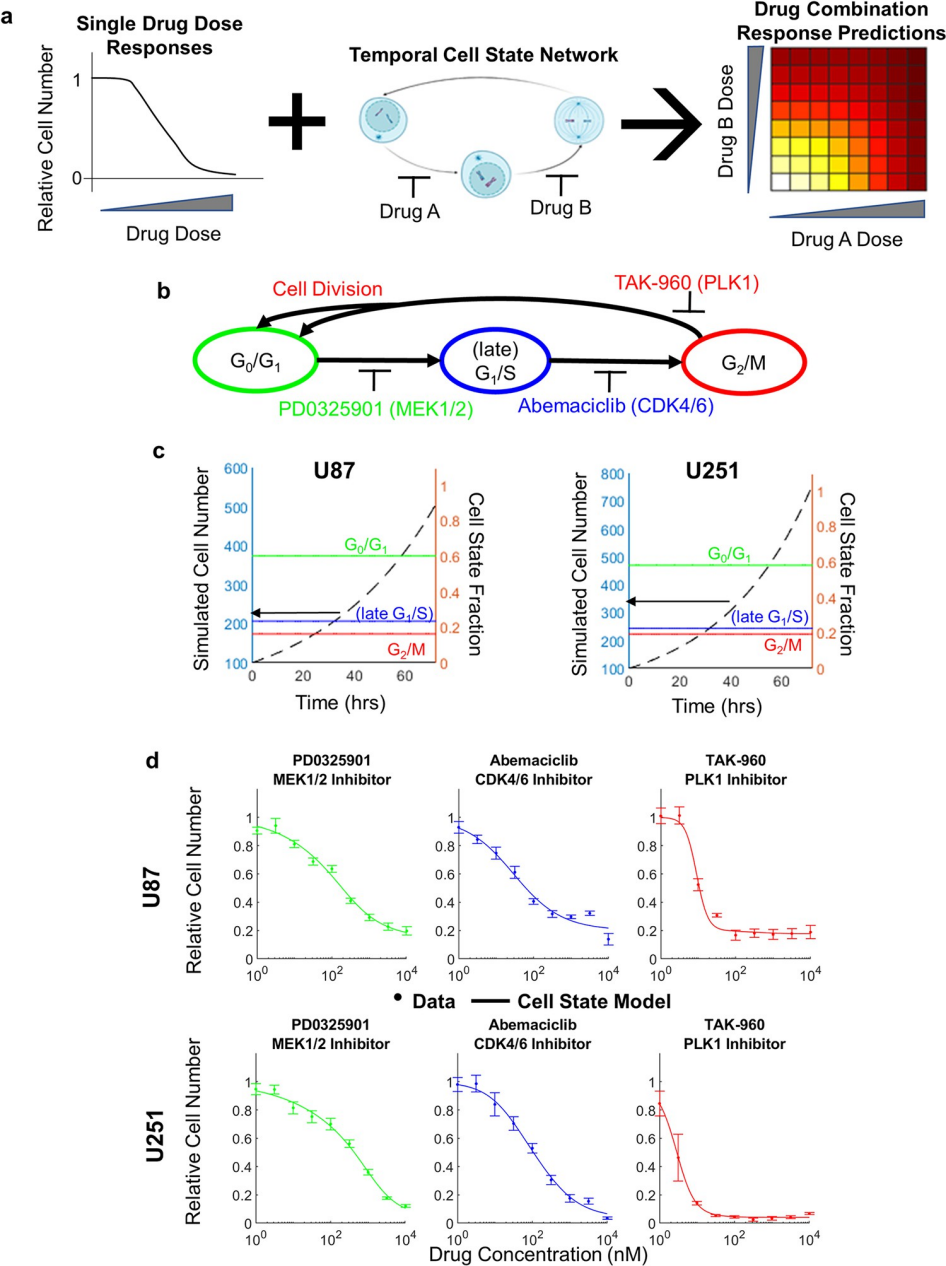
Modeling temporal cell states and single drug dose responses. **(a)** Graphical abstract. Schematic showing the idea that integration of single drug dose response experiments with temporal cell state network models might enable prediction of drug combination responses. **(b)** Schematic of the temporal cell state network comprising G0/G1, late G1/S and G2/M states and the activity of the drugs PD0325901, Abemaciclib and TAK-960 in each state. In this work, we assume that the action of each drug is specific to the state indicated by color. Cells exiting G2/M divide when they re-enter G0/G1. **(c)** Time courses of cell number in the temporal cell state model for U87 and U251 cells starting with 100 cells for 72 hours. Cell proportions at G0/G1, late G1/S and G2/M states are also shown, which remain constant. **(d)** Single drug dose responses for PD0325901, Abemaciclib and TAK-960 in U87 and U251 cells at 72 hours (points) compared to model predictions (lines). Error bars denote standard error.

To test our hypothesis that knowledge of single drug dose responses combined with cell state transition network dynamics could enable prediction of drug combination responses **(Fig 1A),** a model system is needed. There are a variety of choices for cell state transition networks and drugs which modulate them; here we focus on the cell cycle and three targeted kinase inhibitors (**Fig 1B**). Specifically, we focus on a MEK1/2 inhibitor (PD0325901) that primarily blocks transition of G_0_/G_1_ cells [49], a CDK4/6 inhibitor (abemaciclib) that primarily blocks transition of (late)G_1_/S cells [50–53], and a PLK1 inhibitor (TAK-960) that primarily blocks transition of G_2_/M cells [54–57]. Drug dose response experiments evaluating cell number after 3 days of treatment show that both U87 and U251 cells are responsive to these drugs as single agents (**Fig 1D**). These cell lines and drugs were chosen as a subset of a larger ongoing project; however, the hypothesis under investigation is agnostic to the particular cell lines and drugs chosen. Wider application beyond this initial set would enable more extensive evaluation of how substantial differences between cell lines and drugs impact the hypothesis.

Before accounting for drug effects, we first constructed and parameterized a temporal cell state network model based on Markov formalisms that describes cell population growth dynamics in the absence of drug for U87 and U251 cells. The reader is referred to the Methods section for full details of model assumptions, development, and parameter estimation. Cells in the G_0_/G_1_ state can transition to the (late)G_1_/S state, which can then transition to the G_2_/M state. Upon transition from G_2_/M to G_0_/G_1_, cell division occurs, increasing cell number by one. For this case without drug, we consider cell death transitions (which decrease cell number by one) to be negligible. In each time step (chosen to be 1 hr), cells can either remain in their current state, or transition. We estimated the three unknown transition probabilities for each cell line by requiring agreement with population doubling time (31.13 hours-U87 [58–60] and 24.93 hours-U251 [61–63] and the steady-state cell state ratios (60:24:16 for U87 [64–68] and 58:23:19 for U251 [69–71]) (**Fig 1C and Table 1**). Simulations recapitulate these features. There is variability reported in the literature for the doubling time and steady-state cell state ratios; below we evaluate the effects of this variability on drug combination response predictions.

Drug action was modeled by assuming the transition probabilities are a sigmoidal function of drug dose (see Methods). Biologically, we assume that PD0325901 blocks transition of G_0_/G_1_ cells [49], Abemaciclib blocks transition of (late)G_1_/S cells [50–53], and TAK-960 blocks transition of G_2_/M cells [54–57]. Fitting to the single drug dose response data yielded reasonable agreement between model and data for most drug doses, but with some systematic variation at high drug doses (**Fig A in S1 Text**). The reason for this systematic variation was that cell counts for high drug doses were lower than the initial cell count, implying cell death occurred. Therefore, we allowed the drugs to induce cell death (see Methods). This refined model could account for responses at high drug doses with improved agreement overall (*r*~0.99, ΔAIC = -160.1, **Fig 1D and B in S1 Text**). Overall, these results demonstrate that the Markov model of cell state transition dynamics can capture cell population growth and single drug responses for the investigated system.

**Fig 2:**
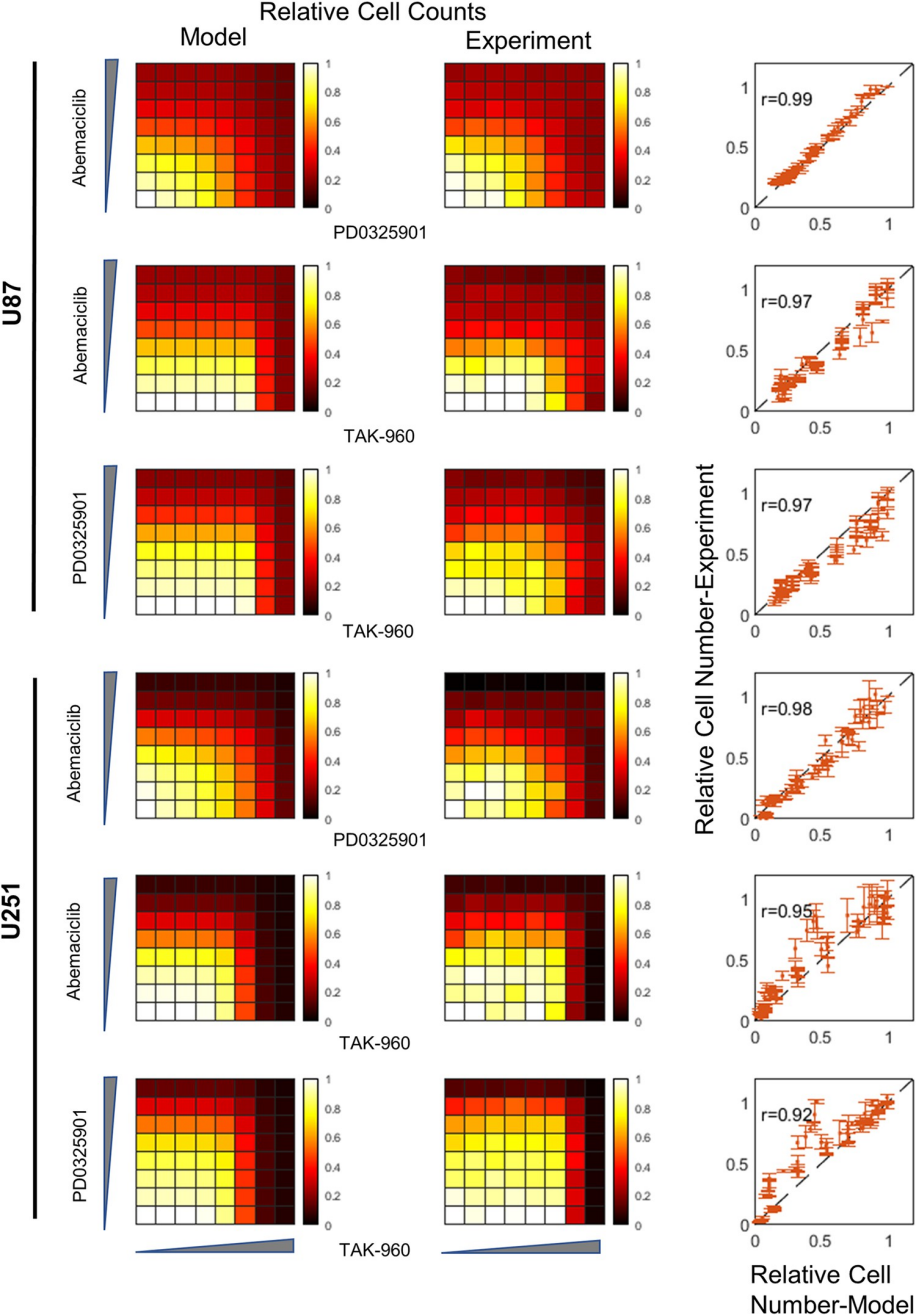
Model prediction vs experiments for drug combination responses. Predicted and measured combination drug dose responses for Abemaciclib/PD0325901, Abemaciclib/TAK-960 and PD0325901/TAK-960 for U87 cells (top) and U251 cells (bottom). First column is relative cell counts for model simulations, second column is relative cell counts for experiments, and the third column is a scatterplot for model vs experiment for relative cell counts. The drug concentrations (nM) for Abemaciclib and PD0325901 are 0, 1.22, 4.88, 19.53, 78.13, 312.5, 1250, and 5000, and for TAK-960 are 0, 0.012, 0.049, 0.20, 0.78, 3.13, 12.5, and 50. The correlation coefficient for agreement between model and experiment for each cell line/drug combination pair is indicated. Error bars denote standard error.

Now that we had a model that could take as input any of the three drugs at any dose and simulate cell population dynamics, we could predict how drug combinations would affect cell number for all pairwise combinations of the three drugs (**Fig 2, left**). These predictions demonstrate reasonable agreement with independent experimental data for every drug combination for each cell line (*r*~0.92–0.99; **Fig 2-middle and right**). The largest discrepancies were evident for TAK-960, particularly for U251 cells (**Fig 2-bottom two rows)**. These discrepancies seem to be predominantly due to experiment showing decreased sensitivity to TAK-960 as a single agent relative to the model (lighter color further to the right). This may be a result of (i) the steep dose response of these cells to TAK-960 (*n>1*, **Table 2**) combined with (ii) finer dose ranges used in the combination experiments around the responsive ranges (see Methods and Fig 2 legend), leading to greater uncertainty in these dose ranges based only on the single drug responses. Notably, no modifications were made to the model—only information about the cell state transition network dynamics and single drug dose responses were needed to perform this prediction. We evaluated the ability of the model to account for drug combination response data when the input doubling time and cell state ratio data were varied between maximum and minimum reported values (**Fig C in S1 Text**). The agreement between model and data was robust to this variation (r~0.92–0.99, similar to previous).

**Fig 3:**
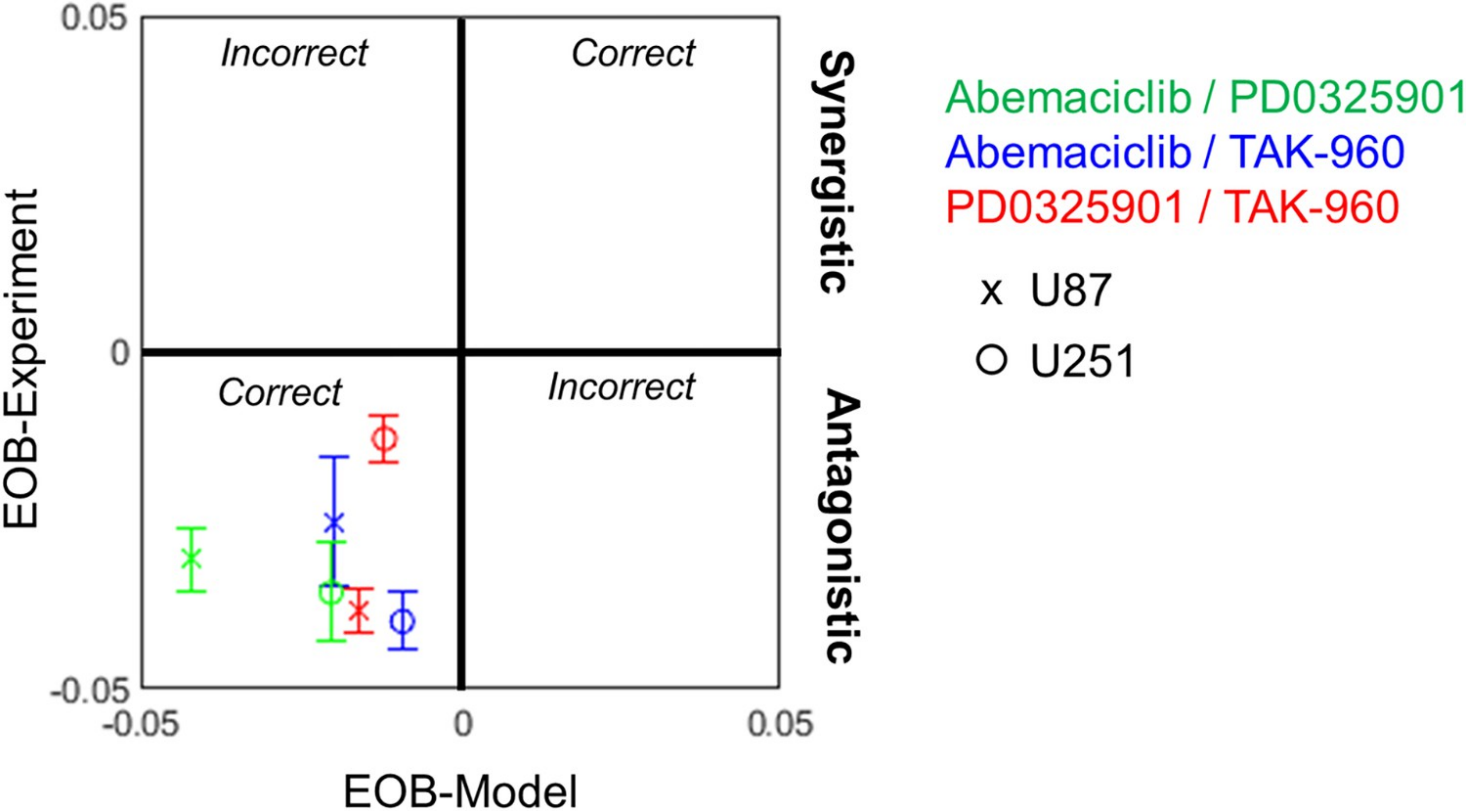
Excess Over Bliss Analysis. Excess over Bliss (EOB) for each drug combination dataset in simulations and experiments were calculated as described in Methods, and mean summarized to obtain as single EOB score for each cell line / drug combination pair. Positive scores denote synergy while negative scores denote antagonism. Error bars are standard error.

Analysis of drug combination responses often includes assessment of drug synergy or antagonism, a more qualitative and categorical analysis. A common analysis is excess over Bliss (EOB) [72], which captures how much of the observed drug response is beyond statistically independent action by each drug. In particular, we used a variation of excess over Bliss that is more robust and reproducible because it uses sigmoidal fits to data to mitigate the impact of experimental noise in any single data point [73]. Values close to zero indicate non-interacting combinations, positive values indicate synergistic combinations and negative values indicate antagonistic combinations. We calculated EOB for model and experiment, yielding an averaged single score for each drug combination / cell line pair, and evaluated agreement between the two (**Fig 3**). Both model and experiment show drug combinations were predominantly mildly antagonistic or non-interacting with co-localization in the bottom left quadrant. Overall, these results provide support for the hypothesis that responses to anti-cancer drug combinations can be predicted with a model of cell state transition dynamics and knowledge of single drug dose responses, for single cell lines *in vitro*.

**Table 1:** Best Fit Markov Transition Parameters.

	U87	U251
*M*_*1*_ *(hr*^*-1*^*)*	0.05	0.07
*M*_*2*_ *(hr*^*-1*^*)*	0.11	0.15
*M*_*3*_ *(hr*^*-1*^*)*	0.14	0.15

**Table 2:** Best Fit Drug Response Parameters.

	PD0325901	Abemaciclib	TAK-960
***U87***			
*EC*_*50*_ *(μM)*	0.10	0.01	0.0073
*n*	0.50	0.65	2.88
*E*_*max*,*φ*_ *(hr*^*-1*^*)*	0.011	0.0028	0.0025
*EC*_*50*,*φ*_ *(μM)*	0.40	0.033	0.28
***U251***			
*EC*_*50*_ *(μM)*	0.56	0.19	0.0025
*n*	0.40	0.76	1.69
*E*_*max*,*φ*_ *(hr*^*-1*^*)*	0.034	0.026	0.022
*EC*_*50*,*φ*_ *(μM)*	2.77	0.042	0.0070

## Discussion

Predicting how varied drug combinations control cancer cell population growth is key to improving cancer precision medicine. Experimental solutions alone cannot cover the vast combinatoric space comprising drug combinations and different cancer cell types, necessitating computational approaches. Any computational approach should rely only on data that is available and/or feasibly attainable. Here, we explore the use of a computational approach that, rather than focus on biochemical networks in which drug targets reside, focuses on cell state networks where drugs influence transitions. By combining information about the cell state network dynamics with single cell drug dose responses, we were able to predict combination responses for three different targeted anti-cancer drugs in two different cell lines with no additional model modifications, with reasonable agreement between model and experiment (*r*~0.92–0.99). We expect this finding to be impactful to inform expansion to different drugs, cell types, and more complex biological systems such as co-culture or *in vivo* models.

The drugs studied here were found to be mildly antagonistic and have not been evaluated as combinations in other studies to our knowledge, including large-scale screening studies [38,74,75]. Interestingly, these previous large-scale screening studies suggest most drug combinations are non-interacting or antagonistic. The fact that the particular drugs studied were found to be antagonistic could be interpreted as a consequence of sequential action on different cell cycle stages, with treatment of one stage resulting in fewer cells available to respond to other drugs, similar to a classical interpretation of negative cooperativity. Yet, a general theory for understanding how cell state or drug target network structure gives rise to drug combination behavior is not yet established, and future studies on this topic would be beneficial.

Combination therapy for the analogous drug targets of MEK1/2, CDK4/6, and PLK1 have been reported but paint an incomplete and inconsistent picture, which may be due to the lack of consistent experimental and analytical systems for evaluating synergy, as well as the large variation between different tumor and cancer cell line types. In KRAS mutant colorectal cancer, combinations of CDK4/6 and MEK inhibitors have been found to be synergistic in reducing *in vitro* cell growth, and to be effective in patient-derived xenograft models [76]. They were reported to cooperate in pancreatic cancer models as well [77]. The rationale was that resistance to the MEK inhibitor by reactivating the pathway could be blunted by also blocking downstream with the CDK4/6 inhibitor, similar to combined BRAF and MEK inhibition in melanoma [28]. Similarly, multiple cancers that had become resistant to CDK4/6 inhibition were found to be sensitized to MEK inhibition [78]. In a single NRAS mutant melanoma patient, this combination was found to be effective, perhaps related to CDKN2A mutation status [79]. A PLK inhibitor showed synergy with only one of two BRAF (upstream of MEK) inhibitors studied in a panel of melanoma cell lines [80], and demonstrated efficacy in a NRAS mutant melanoma [81]. PLK inhibitors were shown to be effective in ER-positive breast cancers that were resistant to CDK4/6 inhibition [82], however other studies showed antagonism in pancreatic cancer [77].

While this study provided support for the idea that drug combination response could be predicted from knowledge only of cell state networks and single drug dose response, it was limited to single cell lines *in vitro*, and only to three drugs and two cell lines. An obvious next step is scaling tests to more single cell lines and drugs. We expect that such scaling would reveal more difficult scenarios where a single drug may target multiple cell state transitions, or where multiple drugs may target the same cell state transition. New methodology would be needed to specify and constrain such mapping; mechanistic modeling of drug target networks could be of use here [22–26,38,41,83].

A single cell line *in vitro* is an extremely simple situation that neglects the impact of, for example, multiple non-tumor cell types, different tumor cell subclones, 3D environments, non-exponential (e.g. Gompertz-like) growth, and long-term development of drug resistance. More cell fates such as apoptosis (and variants such as ferroptosis and necroptosis), senescence, and movement should be considered. The model employed here also does not consider spatial phenomena that could influence cell-cell communication, both physical and chemical. The presence of multiple subclones and non-cancer cell types within a tumor could create cell populations and niches with different drug or immune sensitivities. Multiple agent-based modeling approaches have been described that are suitable for capturing these important phenomena [84–88]. Evolutionary dynamics on larger time scales are critical to capturing resistance, a major focus of combination therapy. Ecological and game theory models have been proposed to describe such effects, by proposing “adaptive therapy” regimens that intelligently (and in a sense optimally) switch between drugs to maintain tumor burden at manageable levels [89–93]. These types of models seem in principle compatible with the models and ideas described in this paper.

Pharmacokinetics are another important aspect that remains to be explored. Drugs are often given in different temporal sequences but here we only investigated simultaneous administration. The proposed cell state network models do have the ability to explore drug temporal sequence, and this is straightforward to implement with *in vitro* experiments, so this is a logical next step for exploring how to better administer drug combinations. For eventual *in vivo* situations, drug concentration is not constant, but again, the dynamic nature of the cell state models is amenable to handling such changes. Lastly, drug concentration is not constant across spatial dimensions of the tumor. Integration with existing partial differential equation or agent-based models could help account for spatially-varying drug concentration [84–88].

While we do not explicitly consider the role of biochemical networks in drug combination response, in a sense, they are implicitly accounted for in the mapping of drug concentration to cell state transition rates. In the investigated case, there was an arguably clean mapping of drug concentration to single transition rates, which simplified the effort. In other cases, such mapping may not be known *a priori* and/or more complex, i.e., a single drug may influence multiple transition rates, or two drugs may impact the same transition rate. Biochemical network models that capture such complexities or mapping may prove useful in such situations [22–26,38,41,83]. Assumptions regarding the additivity (or not) of multi-drug action on transition probabilities would have to be asserted. The current availability of drug combination response data sets [74,75,94,95] could facilitate the testing of such methods. Such future work could explore drug combination features we did not consider here, such as combining drugs that are not effective as single agents. They could also explore conditions that lead to drug combination synergy; the systems chosen here exhibited predominantly antagonistic behavior. Avoiding antagonism, however, is likely an important goal. It is thought that a small fraction of all drug combinations lead to synergistic behavior [38,74,75], but finding them, and how synergy is controlled by cell type, is of critical importance for precision oncology.

Application of this approach to other systems requires identification of cell state network models. Again, in our case the cell cycle is well established in terms of cell state network structure, but other such networks may not be. For example, gliomas are thought to comprise a network of four fundamental subtypes that can transition between each other, and whose differential growth and drug response characteristics are important for predicting response to therapy [96]. Studies have also confirmed the factors behind certain other cell state transitions-for instance, the transcriptomic factors and signaling molecules in different epithelial to mesenchymal transitions [26,97–99]. Cell state transition networks have been identified for multiple cancer types [11–15,17,18,100–102], generally by combining single cell measurements (e.g. single cell RNAseq), with perturbation time courses, such as enriching for one cell state and then observing the fractional composition dynamics. Recently, we proposed a general theory built upon modular response analysis [103–106] that allows one to reconstruct cell state networks from such perturbation time course data [107]. This theory is compatible with the Markov formalisms used here. Such Markov formulation may have further applicability to other cell state systems [18,101,102,108–110], but other approaches have been used [111–114]. Cell state transitions are subject to inherent stochasticity and describing the cell transitions as a Markov process is a common tool to capture this probabilistic aspect. However, this also relies on the assumption that the transition probabilities and the underlying variables are known and that the cell states are properly sampled and well classified. There could be several knowledge gaps in these assumptions including that cells may be transcriptomically intermediate between canonically defined states [115] and biological data may be sparse [116]. Inclusion of methods such as lineage tracing and methods able to handle sparse data [113] may help address some of these gaps.

Overall, we have tested a relatively simple hypothesis that knowledge of single drug dose responses combined with cell state network dynamics is sufficient for prediction of drug combination responses. This hypothesis seems to hold true at least for the three drugs and two cell lines tested here *in vitro*, providing a potentially powerful rationale for guiding drug combination response modeling efforts. Expansion to more cell lines, cell state systems, and drugs will of course be important for further testing. Our findings here provide an important step towards being able to predict how cancer cell populations will respond to combinations of anti-cancer drugs, a key capability for cancer precision medicine.

## Methods

### Experimental Methods

#### Cell culture

U87 and U251 cells (both STR verified) were cultured in full growth medium comprising DMEM (Gibco #10313039) supplemented with 10% FBS (Corning #35-011-CV) and 2 mM L-Glutamine (Corning #25–005-CI). The cells were cultured at 37°C in 5% CO_2_ in a humidified incubator and passaged every 2–3 days with 0.05% trypsin (Corning #25–052-Cl) to maintain sub-confluency.

#### Single drug dose response experiments

U87 and U251 cells were seeded in 96 well plates (Corning-Falcon #353072) with 500 cells per well, counted with a hemocytometer. Cells were seeded in 90 μl full growth media and cultured overnight. The next day, 10 μl of media containing 10X the final drug concentration was added and the plates cultured for 72 hours. Experiments were done in biological duplicate with technical triplicates for each.

The three drugs were procured from the following sources—PD0325901 (Selleckchem #S1036), Abemaciclib (Selleckchem #S5716) and TAK-960 (Tocris #5403). The quantities of each drug-PD0325901 (25 mg, molar mass-482.19g), Abemaciclib (25 mg, molar mass-506.59g) and TAK960 (10 mg, molar mass-598.06g) corresponded to 0.0518 millimoles, 0.0493 millimoles and 0.0167 millimoles respectively and were diluted in 5.18 mls, 4.18 mls and 1.67 mls of sterile filtered DMSO to bring the final concentration to 10 mM for each drug. These dilutions were then aliquoted into 10 μL batches. Before adding to cells, 990μL of full growth media was added to a 10 μL drug aliquot, diluting it to 100 μM, 10X times the highest desired dose. This concentration was further serially diluted 8 more times in full growth media containing 1% DMSO (to maintain the same DMSO concentration in each dilution) and by a factor of 3.16 each time. This results in 9 dilutions with the drug concentrations between 10 nM-100 μM. In 9 wells with cells seeded overnight in 90 μL media, 10 μL of the serially diluted drugs are added. In the 10^th^ well, 10 ul of full growth media containing 1% DMSO was added as the vehicle control dose.

#### Drug combination response experiments

U87 and U251 cells were seeded in 96 well plates (Corning-Falcon #353072) with 500 cells in each well. Eight by eight wells were seeded in 150 μl full growth media and cultured overnight. The next day, 25 μl of media was added twice to each well, each containing 8x of the final drug concentration and cultured for 72 hours. The final drug concentrations were chosen to reflect their responsive range for the cell lines (1.22 nM-5μM for PD0325901 and Abemaciclib, 0.0122nM-50nM for TAK960, with a factor of 4 spacing). Before adding the drugs to cells, as above, full growth media was added to a 10 μL drug aliquot, diluting it to 8X times the highest desired dose. This concentration was further serially diluted 6 more times in full growth media containing 1% DMSO (to maintain the same DMSO concentration in each dilution) and by a factor of 4 each time. Experiments were done in biological triplicate.

#### Staining and computational image analysis

After 72 hours of treatment with the drugs, the cells were stained with Hoechst (BDBiosciences #BD 561908) and Propidium Iodide (Millipore Sigma #P4170) at a final concentration of 1 μg/ml and 2 μg/ml to stain all cells and dead cells, respectively. After 30 minutes, the wells were imaged using the TagBFP (Excitation-390/18 nm, Emission-447/60 nm) and RFP filters (Excitation-531/40 nm, Emission-593/40 nm) in a Cytation 5 (Biotek). Each image was flatfield corrected and background subtracted using CellProfiler. The nuclei were then identified using the IdentifyPrimaryObjects feature, and a pseudo image was generated. The number of all counts of cell nuclei stained with Hoechst and Propidium Iodide were compiled by CellProfiler and exported as csv files (provided in the code). The Propidium Iodide-stained nuclei counts were subtracted from the Hoechst stained nuclei counts for each well, and this was taken as the live cell counts which were the primary data.

### Computational methods

#### Biological assumptions

We assume that cells in the G_0_/G_1_ state can transition to the (late)G_1_/S state, which can then transition to the G_2_/M state. Upon transition from G_2_/M to G_0_/G_1_, cell division occurs, increasing cell number by one. Without drug, we consider cell death transitions (which decrease cell number by one) to be negligible. In each time step (chosen to be 1 hr), cells can either remain in their current state, or transition. Drug action was modeled by assuming the transition probabilities are a sigmoidal function of drug dose (see below). PD0325901 blocks transition of G_0_/G_1_ cells [49], Abemaciclib blocks transition of (late)G_1_/S cells [50–53], and TAK-960 blocks transition of G_2_/M cells [54–57]. We allow for each drug to also induce cell death since a model without this could not account for data at high drug dose (**Fig A in S1 Text**).

#### Markov model of temporal cell state transitions

Consider a Markov transition model comprising three nodes, representing cell states G_0_/G_1_, late G_1_/S and G_2_/M, denoted 1, 2, and 3 in short. *M*_*1*_, *M*_*2*_ and *M*_*3*_ are the proportions of cells transitioning from states 1–2, 2–3 and 3–1 respectively, within a given timestep, taken to be one hour. *M*_*11*_, *M*_*22*_ and *M*_*33*_ are the proportion of cells that do not transition from states 1, 2 and 3 respectively, in that hour. A cell in state 3 undergoes cell division which gives rise to two cells in state 1. We formulate this scenario using a jump Markov process model as follows:

$$x_{1,t+1}=M_{11}x_{1,t}+2\times M_{3}x_{3,t}$$

$$x_{2,t+1}=M_{22}x_{2,t}+M_{1}x_{1,t}$$

$$x_{3,t+1}=M_{33}x_{3,t}+M_{2}x_{2,t}$$ (1)

where, *x*_*i*,*t*_, are the numbers of cells in state *i* at time point *t*. We set the time interval between two Markov jumps at 1 hour and simulate the model for a total of 72 hours.

These equations are subject to the constraints that the proportion of cells within a state must add to 1. Therefore,

$$M_{ii}+M_{i}=1$$ (2)

or

$$M_{ii}=1-M_{i}$$ (3)

Incorporating this in the above equation enables representation of the system in terms of *M*_*1*_, *M*_*2*_ and *M*_*3*_ only:

$$x_{1,t+1}=(1-M_{1})x_{1,t}+2\times M_{3}x_{3,t}$$

$$x_{2,t+1}=(1-M_{2})x_{2,t}+M_{1}x_{1,t}$$

$$x_{3,t+1}=(1-M_{3})x_{3,t}+M_{2}x_{2,t}$$ (4)

In short this may be represented as follows

$$x_{i,t+1}=(1-M_{i})x_{i,t}+f\times M_{j}x_{j,t}$$

ifi=1:f=2,j=3

ifi=2,3:f=1,j=i−1 (5)

We estimated the unknown transition rate parameters *M*_*1*_, *M*_*2*_, and *M*_*3*_ based on experimental data for cell doubling times of 31.13 and 24.93 hours and cell state ratios (G0/G1:G1/S:G2/M) of 0.602:0.235:0.163 and 0.581:0.225:0.194 for U87 and U251 cells respectively (data references in Results). For U87, max/min values were 37.1/25.5 and 0.668:0.263:0.069 / 0.54:0.23:0.23. For U251 max/min values were 27.8/23.0 and 0.716:0.204:0.08 / 0.482:0.237:0.281 (Fig C in S1 Text). We used the doubling time (*τ*_*d*_) to estimate how many cells should be present after 72 hours starting from 100 cells ($100e^{72*\ln(2)/\tau_{d}}$), and multiplied this number by the cell state ratios to estimate *x*_*1*,*72*_, *x*_*2*,*72*_, and *x*_*3*,*72*_. We used fmincon in MATLAB with a least squares formulation to estimate *M*_*1*_, *M*_*2*_ and *M*_*3*_ based on these constraints. We repeated this estimation 5 times with random initial guesses, each converging to the same values, demonstrating uniqueness of the estimates. The best fit values are given in Table 1 below.

#### Drug dose response modeling

Each of the drugs, PD0325901 (MEK1/2 inhibitor—*i = 1*), Abemaciclib (CDK4/6 inhibitor—*i = 2*), and TAK-960 (PLK1 inhibitor—*i = 3*), were modeled as having an inhibitory effect on the respective Markov parameters *M*_*1*_, *M*_*2*_ and *M*_*3*_. We used a sigmoidal hill-type function to describe the effect of drug concentration on transition rates as follows

$$M_{i}=M_{io}\times (1-\frac{(D_{i}/E{C_{50}}_{i}{)}^{n_{i}}}{1+(D_{i}/EC_{50i}{)}^{n_{i}}})$$ (6)

where *M*_*io*_ is the best fit transition parameter from above, *D*_*i*_ is drug dose, and unknown parameters are *EC*_*50i*_ and *n*_*i*_.

To model the effect of drug on cell death and account for data at high drug doses, we added the following term to the model

$$M_{i,\varphi }=\frac{E_{\max i,\varphi }(D_{i}/EC_{50i,\varphi })}{1+(D_{i}/EC_{50i,\varphi })}$$ (7)

where, *E*_*maxi*,*φ*_ and *EC*_*50i*,*φ*_ are parameters to be estimated as above, with Eq 5 changing as follows:

$$x_{i,t+1}=(1-M_{i}-M_{i,\varphi })x_{i,t}+f\times M_{j}x_{j,t}$$

ifi=1:f=2,j=3

ifi=2,3:f=1,j=i−1 (8)

We used lsqnonlin in MATLAB to estimate these unknown parameters by minimizing the sum of squared errors between model predicted and measured relative cell counts at 72 hours post drug treatment. The best fit parameters are summarized in Table 2 below. AIC calculations for cases of death (24 free parameters) or no death terms (12 free parameters) assumed standard normal distributed errors (residuals normalized by experimental standard deviation). We used normpdf in MATLAB to calculate likelihood function contributions.

#### Excess over Bliss analysis

We used a robust excess over Bliss (EOB) [73] as the basis for these calculations. Consider each row in the combination drug dose response matrix. One of the drug’s doses would be constant across the row but the dose of the other drug increases from left to right. This can be captured by a 4-parameter logistic model [73,117]-

$$y=\frac{E_{\min}+E_{\max}{\left(D/EC_{50}\right)}^{n}}{1+{\left(D/EC_{50}\right)}^{n}}$$ (9)

where *y* is the inhibition effect (1—the measured relative cell number), *E*_*min*_ is the minimal possible inhibition effect, *E*_*max*_ is the maximum possible inhibition effect, *EC*_*50*_ is the half maximal parameter and *n* is the Hill coefficient.

We used lsqcurvefit in MATLAB to obtain least-squares estimates for the four-parameters for each of the 8 rows and 8 columns. For a particular drug combination point, the average of the two fitted inhibition values from its row and column estimates was taken as the final fitted inhibition value (*y*_*AB*_). Consider the fitted inhibition values for particular doses of drug A alone (*y*_*A*_), drug B alone (*y*_*B*_) and their combination (*y*_*AB*_). The Bliss independence scores [33,72,73] are calculated by

$$y_{Bliss,AB}=y_{A}+y_{B}-y_{A}y_{B}$$ (10)

and the *EOB* scores are calculated by

$$EOB_{AB}=y_{AB}-y_{Bliss,AB}.$$ (11)

These scores evaluated at each dose combination are averaged across the dataset for a single EOB score for each drug combination / cell line pair.

## Supporting information

S1 Code This file contains all the MATLAB code necessary to reproduce the figures and analyses in the manuscript (see also Methods). (ZIP)

S1 Text **Which contains the following Figures: Fig A. Single Drug Dose Response Fits for a Model with No Drug-Induced Cell Death.** The model was fit as described in Methods, but there was no ability for drugs to influence cell death. This model could not account for much of the drug action at high concentrations. **Fig B. Goodness-of-Fit for Single Drug Dose Responses.** For each fit shown in Fig 1D, relative cell number for model simulations was plotted vs experimental data. The correlation coefficient was calculated and is shown within each plot. **Fig C. Goodness-of-Fit for Drug Combination Responses for Varying Ranges of Doubling Time and Cell State Ratios.** The doubling times and cell state ratios for cell lines can vary from lab-to-lab, and we used reported ranges for U87 and U251 cells to perform drug combination response simulations as in the manuscript. The correlation between model simulations and experimental data for drug combination responses is shown, and variation in these parameters has minimal effect. Error bars denote standard error. Correlation coefficients are shown in each plot.
(DOCX)
