# Supplementary material for: Predicting anti-cancer drug combination responses with a temporal cell state network model
Source: PLoS Comput Biol. 2023 May 1;19(5):e1011082. doi: 10.1371/journal.pcbi.1011082 (PMC10174488; doi:10.1371/journal.pcbi.1011082)
Supplement: S1 Text — Which contains the following Figures: Fig A. Single Drug Dose Response Fits for a Model with No Drug-Induced Cell Death. The model was fit as described in Methods, but there was no ability for drugs to influence cell death. This model could not account for much of the drug action at high concentrations. Fig B. Goodness-of-Fit for Single Drug Dose Responses. For each fit shown in Fig 1D, relative cell number for model simulations was plotted vs experimental data. The correlation coefficient was calculated and is shown within each plot. Fig C. Goodness-of-Fit for Drug Combination Responses for Varying Ranges of Doubling Time and Cell State Ratios. The doubling times and cell state ratios for cell lines can vary from lab-to-lab, and we used reported ranges for U87 and U251 cells to perform drug combination response simulations as in the manuscript. The correlation between model simulations and experimental data for drug combination responses is shown, and variation in these parameters has minimal effect. Error bars denote standard error. Correlation coefficients are shown in each plot. (DOCX) [file pcbi.1011082.s002.docx]

**Supporting Information for:**

**Predicting Anti-Cancer Drug Combination Responses with a Temporal Cell State Network Model**

Deepraj Sarmah^1^, Wesley O. Meredith^1^, Ian K. Weber^1,2^, Madison R. Price^1,3^, Marc R. Birtwistle^1,4,*^

^1^Department of Chemical and Biomolecular Engineering, Clemson University, Clemson, South Carolina, USA

^2^The University of Virginia School of Medicine, Charlottesville, Virginia, USA

^3^College of Pharmacy, Medical University of South Carolina, Charleston, South Carolina, USA

^4^Department of Bioengineering, Clemson University, Clemson, South Carolina, USA

*[mbirtwi@clemson.edu](mailto:mbirtwi@clemson.edu)

**Contents**

Fig A in S1 Text. Single Drug Dose Response Fits for a Model with No Drug-Induced Cell Death.

Fig B in S1 Text. Goodness-of-Fit for Single Drug Dose Responses.

Fig C in S1 Text. Goodness-of-Fit for Drug Combination Responses for Varying Ranges of Doubling Time and Cell State Ratios.

**
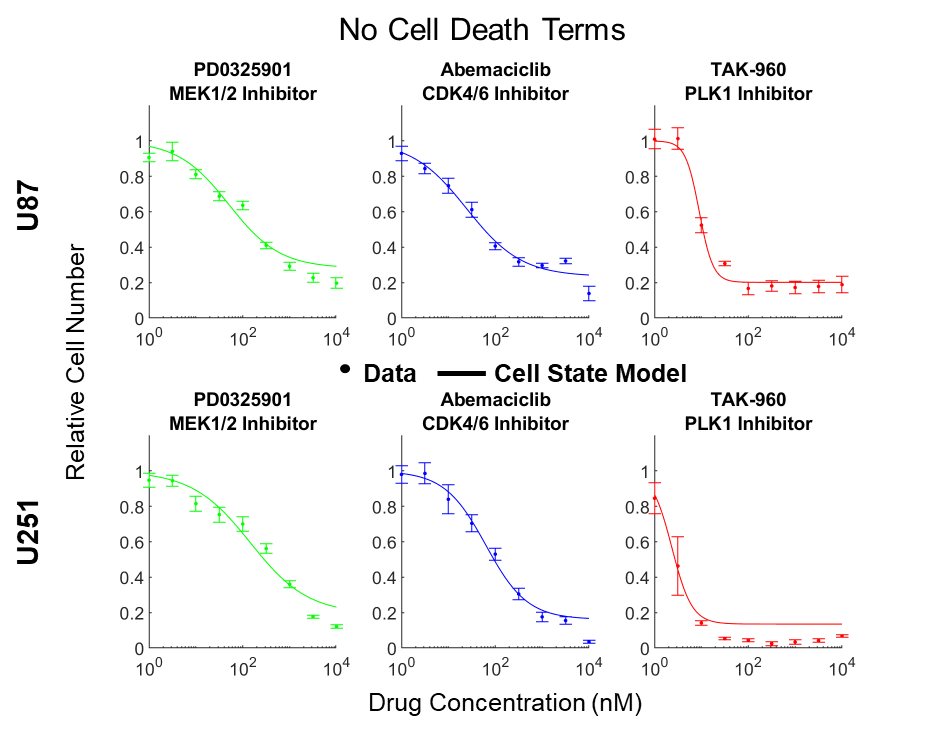
**

**Fig A. Single Drug Dose Response Fits for a Model with No Drug-Induced Cell Death.** The model was fit as described in Methods, but there was no ability for drugs to influence cell death. This model could not account for much of the drug action at high concentrations.

**
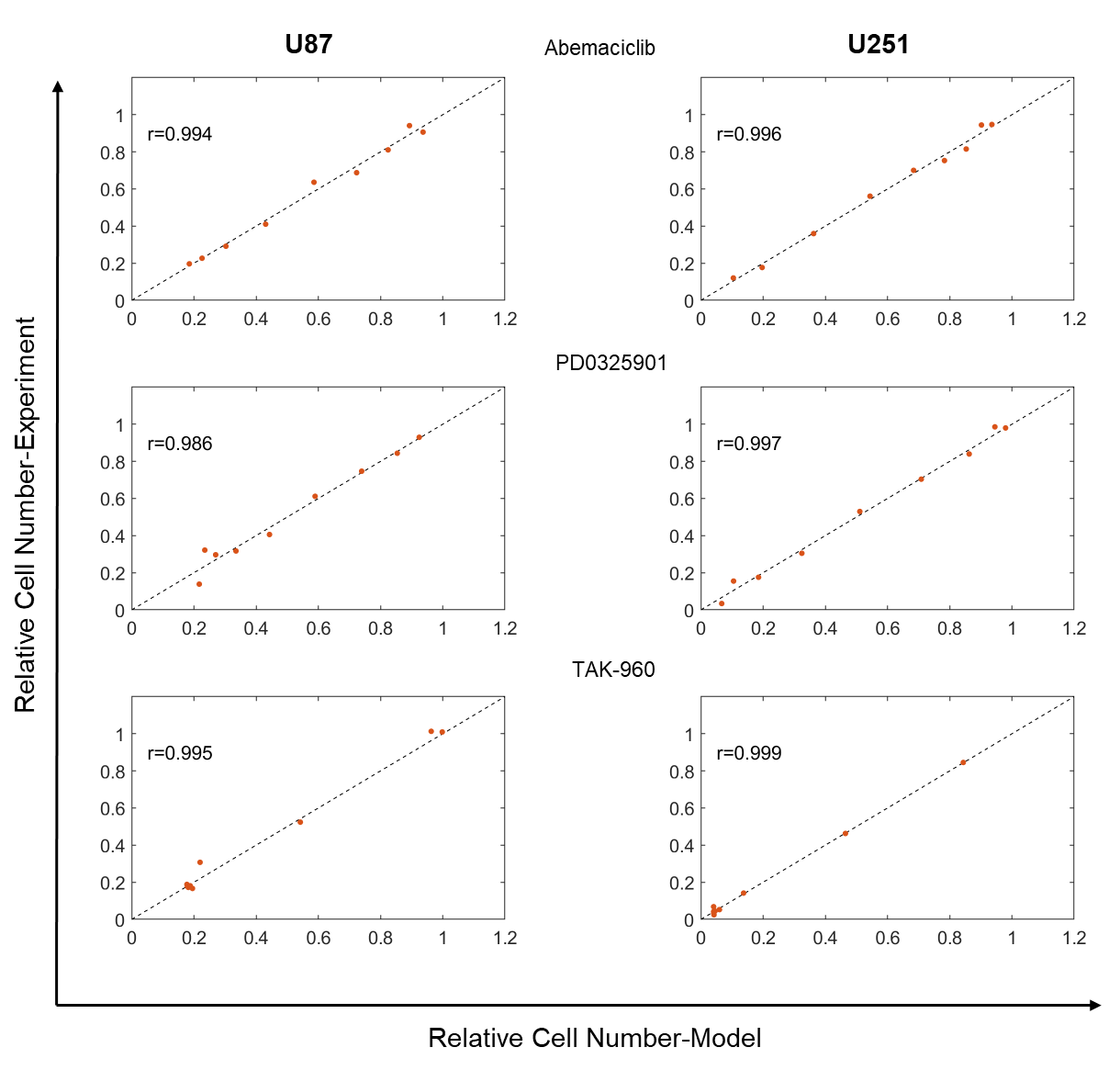
**

**Fig B. Goodness-of-Fit for Single Drug Dose Responses.** For each fit shown in Fig. 1d, relative cell number for model simulations was plotted vs experimental data. The correlation coefficient was calculated and is shown within each plot.

**
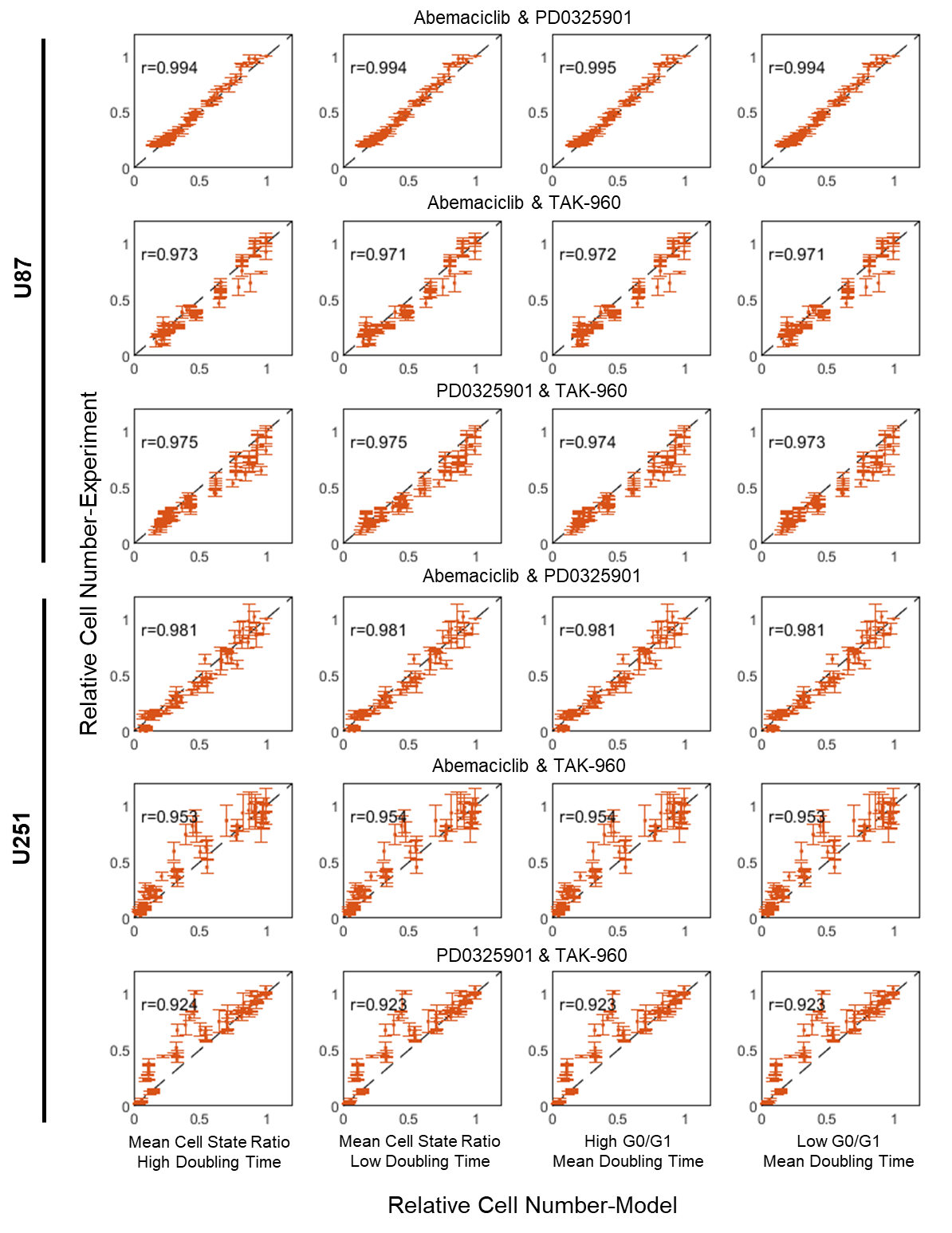
**

**Fig C. Goodness-of-Fit for Drug Combination Responses for Varying Ranges of Doubling Time and Cell State Ratios.** The doubling times and cell state ratios for cell lines can vary from lab-to-lab, and we used reported ranges for U87 and U251 cells to perform drug combination response simulations as in the manuscript. The correlation between model simulations and experimental data for drug combination responses is shown, and variation in these parameters has minimal effect. Error bars denote standard error. Correlation coefficients are shown in each plot.
